# Poster Session II - A216 SECOND-LINE INFLIXIMAB THERAPY FOR CROHN’S DISEASE (SLIT-CD): A RETROSPECTIVE COHORT STUDY

**DOI:** 10.1093/jcag/gwaf042.216

**Published:** 2026-02-13

**Authors:** C Galts, S Anvari, D Borovsky, K Grossman, H R Tran, A Wen, A Albassam, N Habib, A Varghese, S Halder, J Marshall, N Narula

**Affiliations:** Gastroenterology, McMaster University, Hamilton, ON, Canada; Massachusetts General Hospital, Boston, MA; Gastroenterology, McMaster University, Hamilton, ON, Canada; Gastroenterology, McMaster University, Hamilton, ON, Canada; Gastroenterology, McMaster University, Hamilton, ON, Canada; Gastroenterology, McMaster University, Hamilton, ON, Canada; Gastroenterology, McMaster University, Hamilton, ON, Canada; Gastroenterology, McMaster University, Hamilton, ON, Canada; Gastroenterology, McMaster University, Hamilton, ON, Canada; Gastroenterology, McMaster University, Hamilton, ON, Canada; Gastroenterology, McMaster University, Hamilton, ON, Canada; Gastroenterology, McMaster University, Hamilton, ON, Canada

## Abstract

**Background:**

Current North American guidelines position infliximab alongside other first line agents for the management of moderate-to-severe Crohn’s disease (CD). However, data on its efficacy as a second-line agent after advanced therapy remain limited.

**Aims:**

The purpose of this study is to evaluate infliximab’s efficacy as a second-line agent and compare outcomes to first line use.

**Methods:**

We conducted a retrospective cohort study of adults with CD treated with infliximab between 2016-2025 at a Canadian tertiary care hospital. Patients were categorized by timing of infliximab use (bio-naïve/first-line vs experienced). Clinical and endoscopic outcomes were compared at one year.

**Results:**

Among 160 included patients, 123 received infliximab as first line therapy and 37 as second-line or subsequent; of these, 10 received it as third-line or subsequent. The most common reason to change therapy was secondary loss of response. There was no significant difference in disease location between patients who received infliximab as first or second line therapy. Clinical response (70.3% vs 89.4%) and clinical remission (43.2% vs 62.6%) at 1 year were both significantly lower in second-line users (p = 0.036 and p = 0.004, respectively). Of patients with endoscopic data available, response rates were also significantly lower in second-line users (56.5% vs 89.1%, p = 0.001) Second-line users also had significantly higher rates of hospitalization (29.7% vs 6.8%, p = 0.001) and surgery (35.1 vs 12.7%, p = 0.02) compared to first line users. There was no significant difference in immunomodulator use or dose escalation between groups.

**Conclusions:**

Infliximab is an effective treatment for bio-experienced patients with CD, but clinical and endoscopic response rates may be reduced compared to first line users. Further studies are required to determine the optimum positioning and patient selection for infliximab within paradigms of care for CD.

Crohn’s disease one year outcomes comparing first and second-line use of infliximab

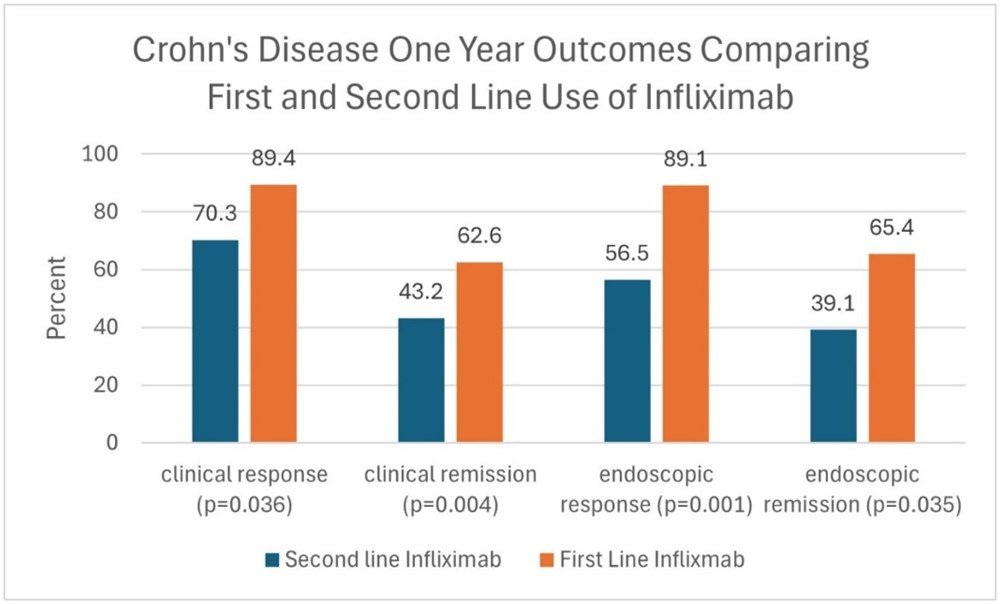

**Funding Agencies:**

None

